# CBGDA: a manually curated resource for gene–disease associations based on genome-wide CRISPR

**DOI:** 10.1093/database/baae077

**Published:** 2024-08-30

**Authors:** Qingsong Du, Zhiyu Zhang, Wanyi Yang, Xunyu Zhou, Nan Zhou, Chuanfang Wu, Jinku Bao

**Affiliations:** Key Laboratory of the State Ministry of Education for Bio-Resources and Ecologic Environment, College of Life Sciences, Sichuan University, 29 Wangjiang Rd, Chengdu 610064, China; Key Laboratory of the State Ministry of Education for Bio-Resources and Ecologic Environment, College of Life Sciences, Sichuan University, 29 Wangjiang Rd, Chengdu 610064, China; Key Laboratory of the State Ministry of Education for Bio-Resources and Ecologic Environment, College of Life Sciences, Sichuan University, 29 Wangjiang Rd, Chengdu 610064, China; Key Laboratory of the State Ministry of Education for Bio-Resources and Ecologic Environment, College of Life Sciences, Sichuan University, 29 Wangjiang Rd, Chengdu 610064, China; Research Center, The Affiliated Brain Hospital, Guangzhou Medical University, 36 Mingxin Rd, Guangzhou 510000, China; Key Laboratory of the State Ministry of Education for Bio-Resources and Ecologic Environment, College of Life Sciences, Sichuan University, 29 Wangjiang Rd, Chengdu 610064, China; Key Laboratory of the State Ministry of Education for Bio-Resources and Ecologic Environment, College of Life Sciences, Sichuan University, 29 Wangjiang Rd, Chengdu 610064, China

## Abstract

The field of understanding the association between genes and diseases is rapidly expanding, making it challenging for researchers to keep up with the influx of new publications and genetic datasets. Fortunately, there are now several regularly updated databases available that focus on cataloging gene–disease relationships. The development of the Clustered Regularly Interspaced Short Palindromic Repeats (CRISPR)-Cas9 system has revolutionized the field of gene editing, providing a highly efficient, accurate, and reliable method for exploring gene–disease associations. However, currently, there is no resource specifically dedicated to collecting and integrating the latest experimentally supported gene–disease association data derived from genome-wide CRISPR screening. To address this gap, we have developed the CRISPR-Based Gene–Disease Associations (CBGDA) database, which includes over 200 manually curated gene–disease association data derived from genome-wide CRISPR screening studies. Through CBGDA, users can explore gene–disease association data derived from genome-wide CRISPR screening, gaining insights into the expression patterns of genes in different diseases, associated chemical data, and variant information. This provides a novel perspective on understanding the associations between genes and diseases. What is more, CBGDA integrates data from several other databases and resources, enhancing its comprehensiveness and utility. In summary, CBGDA offers a fresh perspective and comprehensive insights into the research on gene–disease associations. It fills the gap by providing a dedicated resource for accessing up-to-date, experimentally supported gene–disease association data derived from genome-wide CRISPR screening.

**Database URL**: http://cbgda.zhounan.org/main

## Introduction

The relationship between genes and diseases has been a cornerstone of biological and medical research, illuminating our understanding of pathogenesis and guiding the development of therapeutic interventions. With the exponential growth of research papers in this field over the years, the volume of data has increased tremendously. To illustrate, the number of published articles related to gene–disease associations has grown from 5000 in 2000 to over 60 000 in 2022, reflecting the burgeoning interest and importance of this field of study. Hence, establishing a gene–disease relationship database is of paramount importance as it enables researchers to access and analyze a vast array of information in a unified and structured manner. Such databases serve as invaluable resources for scientists studying specific diseases or genes, facilitating hypothesis generation, experimental design, and the development of novel therapeutic approaches.

Clustered Regularly Interspaced Short Palindromic Repeats (CRISPR) technology has revolutionized the field of genetic engineering and gene editing. This powerful tool allows precise modification of DNA sequences with unprecedented ease and efficiency. The CRISPR-Cas9 system, in particular, has gained immense popularity due to its ability to target specific genes and induce alterations in their sequences [1]. The significance of CRISPR technology lies in its versatility and potential to unravel the complex relationship between genes and diseases. By leveraging CRISPR for whole-genome screening experiments, researchers can systematically identify genes that play crucial roles in disease processes. This approach offers a remarkable opportunity to comprehensively explore the genetic underpinnings of various diseases and discover potential therapeutic targets [2]. Compared to older gene editing techniques such as RNA interference (RNAi), CRISPR technology offers several distinct advantages. RNAi relies on the introduction of small interfering RNAs (siRNAs) to trigger gene silencing, while CRISPR enables precise gene editing by directly modifying DNA sequences. This fundamental difference grants CRISPR the ability to not only silence genes but also introduce specific modifications, including insertions, deletions, and substitutions. Additionally, CRISPR technology is highly efficient, enabling researchers to target multiple genes simultaneously. It also provides greater accuracy and specificity, minimizing off-target effects [3]. These characteristics make CRISPR an invaluable tool for studying gene function and establishing comprehensive gene–disease relationships.

Data derived from CRISPR experiments provide several advantages over other methods of gene–disease association discovery [3, 4]. First, CRISPR enables systematic and unbiased screening of the entire genome, allowing comprehensive identification of genes involved in disease processes. This approach eliminates potential biases associated with candidate gene selection, enhancing the reliability and completeness of the obtained data. Furthermore, CRISPR experiments provide functional validation of gene–disease associations. By precisely manipulating gene expression or function, CRISPR enables researchers to establish causal relationships between genes and diseases, providing valuable insights into disease mechanisms [3, 5].

Several databases, such as Online Mendelian Inheritance in Man (OMIM) [6], DisGeNET [7], and ClinVar [8], have been developed to document gene–disease associations. These databases collect data from different perspectives: OMIM focuses on monogenic diseases, DisGeNET integrates data from several resources to provide a comprehensive view, and ClinVar aggregates information about genomic variation and its relationship to human health [6–8]. However, some limitations exist in these databases, including the lack of information from genome-wide CRISPR screens and the potential for bias due to their reliance primarily on the methods they use to extract data.

In light of these considerations, we developed the CRISPR-Based Gene–Disease Associations (CBGDA) database. CBGDA incorporates critical host gene information identified from genome-wide CRISPR screens for various diseases. The data in CBGDA are manually curated from thousands of articles, ensuring reliability. Following data cleaning, organization, and annotation, the database serves as a platform for elucidating gene–disease relationships, deciphering biological significance, and generating hypotheses. Unlike previous databases, ours is the first to be built upon data derived from genome-wide CRISPR screens. This approach offers several advantages. First, it allows for the unbiased discovery of gene–disease associations as it is not limited by previous knowledge. Then, CRISPR screening can reveal essential genes for a given disease, providing potential therapeutic targets. Finally, it enables high-throughput identification of gene–disease associations, accelerating the pace of discovery.

## Materials and methods

### Article collection

We searched the PubMed database (https://www.ncbi.nlm.nih.gov/pubmed) using the term “Genome wide CRISPR screen OR CRISPR screen” to maximize the collection of potentially relevant articles. Once the relevant literature was obtained, we performed an extensive manual review and data extraction. Specific key details included, but were not limited to, gene symbols, The HUGO Gene Nomenclature Committee (HGNC) IDs [9], cell lines, phenotypes (pro/anti, indicating enhancement or suppression of disease), pathways, and screening detail information (containing specific experimental details of the CRISPR screens). The collected information was organized and integrated to generate a comprehensive dataset.

### Data annotation strategy

When a relationship between a gene and a disease was elucidated in a publication, the gene and disease were incorporated into the dataset. These relationships were bifurcated into two categories: prodisease (gene promoting the disease) and antidisease (gene inhibiting the disease). Based on the narrative of the literature, the process by which a gene promotes or inhibits a disease was summarized under the “function” entry.

Given the variability of CRISPR experimental conditions across different publications, we included “screen type” and “CRISPR library” in the dataset. Positive selection, the process of imposing selective pressure on a cell library already integrated with single guide RNA (sgRNA), enabling only a handful of cells with the target phenotype to survive and enriching key genes, was noted. In contrast, negative selection, where surviving cells do not exhibit the target phenotype, necessitates comparing the abundance of sgRNA at different time points to identify differential sgRNA and key genes. Negative selection can identify genes causing certain functional deficiencies in cells, such as those essential for cell survival with prolonged screening times.

When a study utilized CRISPR screening to identify relevant genes and made preliminary inferences about their relationship with a disease, followed by wet laboratory experiments to validate the reliability of this relationship, “validated” was set to “Y”; otherwise, it was set to “N.” The “evidence” field typically contained the original statements from the paper, which often allowed us to deduce the relationship between the gene and the disease. Minor inconsistencies, like inconsistent gene symbols, were not expected to impact annotation quality and were noted under “remark.”

Furthermore, due to the poor integration of disease classification across different studies and resources, we adopted a direct annotation approach using authoritative disease database and classification system identifiers, including Disease Ontology, Mesh (Medical Subject Headings), MalaCards, OMIM, and International Classification of Diseases (ICD) [6, 10–12].

### External data integration

Our research aims to elucidate the relationship between genes and diseases, with the ultimate objective of unraveling the pathogenesis of diseases and identifying key genetic targets. These targets can potentially be manipulated using specific compounds or drugs to impact the onset or progression of diseases. To facilitate in-depth investigation into these relationships and disease pathogenesis, we not only extracted gene–disease associations from thousands of relevant articles but also annotated the data with information pertaining to chemicals associated with these genes and diseases and their interactions. This comprehensive annotation process enhances the utility of our database, providing researchers with a rich resource to explore the complex interplay between genes, diseases, and potential therapeutic chemicals.

In the field of genomics, genetic variation refers to the hereditary differences at the genomic level between individuals or populations. These variations manifest as DNA sequence differences at specific positions in the genome, known as loci. Most diseases are associated with genetic mutations, which can lead to disease onset when they occur in genes or other functional regions relevant to the disease or disrupt the normal functioning of the genome. For instance, there are single-gene inherited diseases, where a specific disease is caused by a mutation in a single gene. These mutations can be inherited and passed down from parents to offspring [13]. Examples of such diseases include cystic fibrosis, hereditary breast cancer, and hemophilia [14–16]. On the other hand, there are complex, multigene inherited diseases, where multiple gene mutations interact to contribute to disease risk. These mutations may be inherited, but their combination and interaction influence the risk of developing the disease. Examples of complex diseases include cardiovascular diseases, diabetes, and certain types of cancers [17, 18]. To explore the relationship between genes and diseases from the perspective of mutations, we obtained gene mutation data and disease mutation data from the DisGeNET database [7].

Referring to the DepMap (https://depmap.org/portal/) method for displaying gene expression across tumor diseases, we downloaded the OmicsExpressionProteinCodingGenesTPMLogp1 data from this database (https://depmap.org/portal/download/all/?releasename=DepMap+Public+23Q4&filename=OmicsExpressionProteinCodingGenesTPMLogp1.csv). These data include the expression of 19 193 genes in 1450 cell lines (across 74 primary diseases and involving 29 lineages). Based on these data, the expression pattern of each gene searched by users across 74 primary diseases and the expression pattern in different cell lines in each disease were displayed using R package “ggplot2.”

Gene expression data in nontumor diseases were downloaded from the Gene Expression Nebulas (GEN) database (https://ngdc.cncb.ac.cn/gen/) [19]. Finally, read counts and TPM (Transcripts Per Kilobase of exonmodel per Million mapped reads) values for a total of 30 diseases were collected. Differentially expressed genes were detected from read counts using DESeq2 [20].

### Database development and deployment

The front end was developed using HTML5, CSS3 and JavaScript. The layout of every web page was performed using Bootstrap V5 (https://getbootstrap.com/). DataTables was used to display the data in the page (https://datatables.net/). Mol* is a plugin that automatically loads protein structures from the AlphaFold Protein Structure Database [21]. We use it to display interactive 3D protein structures in the detail page of each gene. The Plotly library was used to display statistical pie charts of data in the home page (https://plotly.com/). Cytoscape.js was used to generate a regulation network diagram to put on the web page [22]. Python Django web framework was used for the back end of the site (https://www.djangoproject.com/). The annotated data are stored in a SQLite3 database (https://www.sqlite.org/).

### Supplemental methods

So far, the core data in the database have been extracted from relevant articles within the time period from 2010 to September 2023. All the external data, on the other hand, were extracted from various external resources, such as DisGeNET and GEN, on 30 September 2023. We have set the current version of the database as “version 2309,” which includes both the core data and the external data. We will update the data in the database once a month, including the core data and external data. For the updates of external data, we will download new data from the corresponding sources in the same manner as before at each time point. After manual integration and annotation, the new data will be added to the existing dataset. Currently, for the updates of core data, we will download all relevant newly published literature for each time point. We will manually screen and collect data, annotate them, and then integrate them into the database.

During the data collection process, we have established strict standards for the collection strategy, such as collecting data that have been screened through CRISPR and proven to have a relationship with diseases. Through manual screening of articles and data extraction, we can ensure the reliability and accuracy of the obtained data. In future updates to the database, we will continue to use the current data collection method and explore other improved data mining methods. Our goal is to enhance the efficiency of data collection while maintaining the reliability and accuracy of the data. Many more relevant articles could be retrieved by searching full-text articles in PubMed Central and other resources. Considering this aspect, we will adopt new strategies in the future to cover as many relevant publications as possible. We may still rely on manual retrieval to obtain these literature sources and extract information from them. Additionally, we may explore other methods such as machine learning and text mining in our future work. This will be given due consideration.

## Results

### Data statistics

In our research, we conducted an extensive literature review on the PubMed database, identifying a total of 5778 articles related to whole-genome CRISPR. Through meticulous manual curation, we collected a total of 271 interaction relationships between 241 genes and 52 diseases from those literature studies. There are 187 pairs that promote gene–disease relationships and 84 pairs that inhibit gene–disease relationships. Hepatocellular carcinoma (HCC) has the most related genes, with 34 genes affecting its disease progression, indicating the high heterogeneity of the HCC, while 25 diseases had only one related gene. *MDM2* has the most interacting diseases and plays a promoting role in three diseases. In order to facilitate users to better utilize CBGDA and explore the relationship between genes and diseases collected in this database, we also collected some basic information about those genes and diseases, including the HGNC ID and The Universal Protein (UniProt) ID [23] of the genes from the HGNC database, Disease Ontology ID, disease description, and OMIM number, as well as ICD10, ICD11 number, and class of diseases from the MalaCards database (https://www.malacards.org/) [24]. In addition, information related to CRISPR experiments used by researchers in articles for the gene–disease study is also included in CBGDA, including CRISPR/Cas9 screening types, cell lines, species information, and sgRNA libraries, which could provide easy reference for users.

Genes are distributed across four gene types (Table 1): 209 genes with protein products, 24 microRNAs, 7 long noncoding RNAs, and 1 pseudogene.

**Table 1.** Gene type distribution in CBGDA

Gene type	Count	Percentage
Genes with protein products	209	86.70
Long noncoding microRNAs	7	3
microRNAs	24	10
Pseudogene	1	0.30

**Figure 1.**
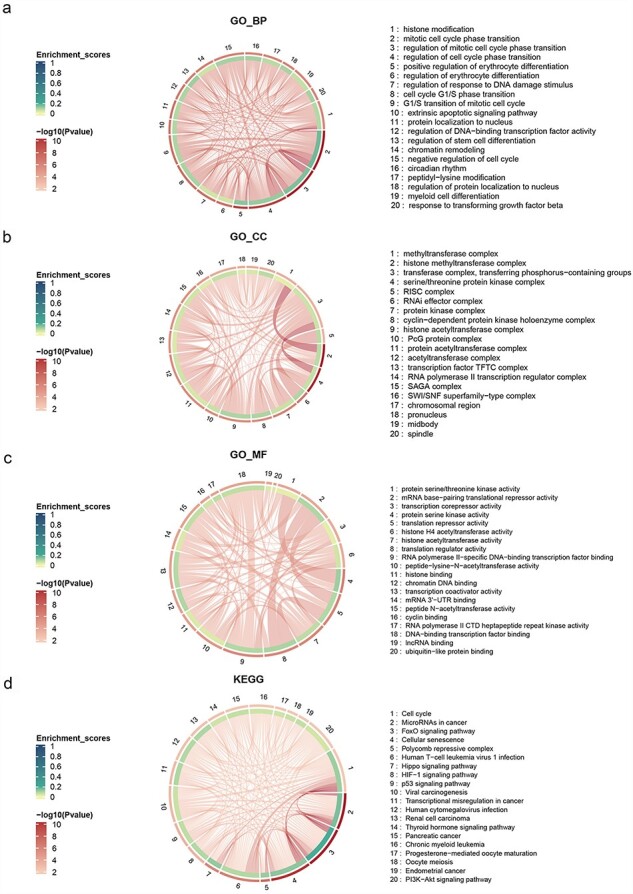
Chord diagram of GO and KEGG enrichment, the outermost layer represents the adjusted *P*-value, the inner layer represents the enrichment ratio, and the strings represent the overlapping number of genes between different enriched terms.

Furthermore, we utilized the GO (Gene Ontology) and KEGG (Kyoto Encyclopedia of Genes and Genomes) enrichment functions of the R package “clusterProfiler” to perform enrichment analysis on 241 genes [25]. These genes were found to be significantly enriched in critical cellular survival pathways and exhibited vital biomolecular functions (*P* ≤ .05) (Table 2 and Fig. 1). The GO enrichment analysis revealed the top five biological processes (BPs) to be histone modification, mitotic cell cycle phase transition, regulation of mitotic cell cycle phase transition, regulation of cell cycle phase transition, and positive regulation of erythrocyte differentiation. The top five cellular components were methyltransferase complex, histone methyltransferase complex, transferase complex, transferring phosphorus-containing groups, serine/threonine protein kinase complex, and RISC complex (RNA-induced silencing complex). The top five molecular functions (MFs) were protein serine/threonine kinase activity, mRNA base-pairing translational repressor activity, transcription corepressor activity, protein serine kinase activity, and translation repressor activity. Furthermore, the KEGG enrichment analysis showed the top five pathways to be cell cycle, microRNAs in cancer, FoxO signaling pathway, cellular senescence, and Polycomb repressive complex. The aberrations in these pathways are intimately associated with disease onset and progression. Therefore, investigating these pathways is certain to advance our understanding of disease progression and facilitate the development of targeted therapeutics.

**Table 2.** Top five terms of KEGG and GO enrichment analysis

Enrichment	Term	Gene count	*P*-value
KEGG	Cell cycle	22	5.57 × 10^−12^
	MicroRNAs in cancer	29	7.42 × 10^−12^
	FoxO signaling pathway	19	6.56 × 10^−11^
	Cellular senescence	17	8.4 × 10^-8^
	Polycomb repressive complex	12	9.22 × 10^-7^
GO			
BPs	Histone modification	36	3.61 × 10^−15^
	Mitotic cell cycle phase transition	32	3.28 × 10^−12^
	Regulation of the mitotic cell cycle phase transition	26	2.7 × 10^−10^
	Regulation of cell cycle phase transition	29	2.7 × 10^−10^
	Positive regulation of erythrocyte differentiation	9	9.79 × 10^−8^
Cellular components	Methyltransferase complex	16	2.8 × 10^−12^
	Histone methyltransferase complex	13	2.46 × 10^−11^
	Transferase complex, transferring phosphorus-containing groups	19	3.33 × 10^-7^
	Serine/Threonine protein kinase complex	12	1.37 × 10^-6^
	RISC complex	20	4.13 × 10^-6^
MFs	Protein serine/threonine kinase activity	23	7.7 × 10^-7^
	mRNA base-pairing translational repressor activity	18	1.41 × 10^-6^
	Transcription corepressor activity	15	1.67 × 10^-6^
	Protein serine kinase activity	20	2.07 × 10^-6^
	Translation repressor activity	18	2.71 × 10^-6^

### Interface description and application

#### Main page

**Figure 2.**
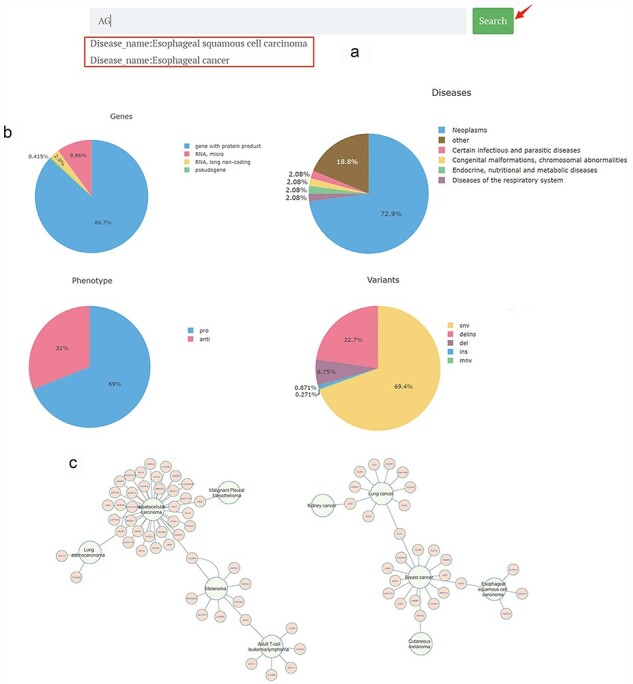
Main page of CBGDA: (a) An example of searching “AG”; (b) Diagrams of statistics of different types of genes, diseases, phenotypes, and variants; (c) Interaction diagram of the associations between genes and diseases.

On the homepage, a brief introduction to the primary functions and potential impact of the CBGDA database is provided. A search bar allows users to input a gene or disease of interest; when a gene or a disease is found within the database, a dropdown menu is triggered; and upon clicking “search,” they are redirected to the detailed page of the respective gene or disease, or if the queried entity is not found, a “Not found” note is displayed (Fig. 2a).

The pie charts visualize the categorization and proportional representation of the core data within the database, including genes, diseases, mutation data, and gene–disease relationship data. Hovering the cursor over each section of the pie chart displays the corresponding category’s name and quantity (Fig. 2b).

The interaction diagram presents a network interaction diagram of the gene–disease relationships in the database. For instance, HCC was found to be associated with the highest number of genes, with 34 genes impacting its disease progression, indicating the high heterogeneity of HCC. In contrast, 25 diseases were found to have only one related gene (Fig. 2c).

The navigation bar permits browsing of the core data information in the database via the “Browse” option. The “HELP” button provides a manual to assist database users in understanding the meaning and usage of each term. The “CONTACT” option enables users to contact the author.

#### Browse

**Figure 3.**
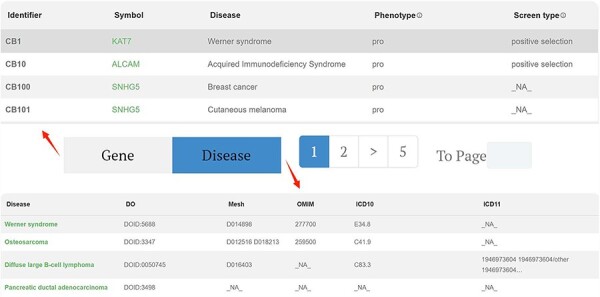
Browse page of CBGDA: The core data of the database using a tabulated format, with pagination buttons to segregate gene data and disease data.

We present the core data of the database using a tabulated format, with pagination buttons to segregate gene data and disease data. (Fig. 3) In the gene data section, each row represents a gene–disease relationship identified from a publication. The displayed data include the gene symbol, disease name, phenotype, type of CRISPR screening, evidence, and PubMed Unique Identifier, among other parameters. The specific explanation for each column header is provided in the “HELP” interface.

By clicking on the “Disease” tab, the user can see the information for all retrieved diseases, including the disease description and Disease Ontology database ID, among others. This feature facilitates users in searching for the disease across different databases. In both the gene data and disease data sections, clicking on the gene name or disease name in the first column redirects the user to the corresponding detailed page.

For instance, in the first column of data, researchers have identified, through genome-wide CRISPR screening, that the gene *KAT7* promotes the onset and progression of Werner syndrome. This specific functional pathway is detailed in the “Function” column. The CRISPR screening employed positive selection, utilized the GeCKOv2 library, and was conducted on the hMPC cell line. This conclusion was further substantiated through additional experimental validations.

#### Gene detail page

We delineate the detailed gene page, which is accessible by clicking either “search” in the homepage search bar or the gene symbol in the table on the “Browse” page. For instance, we illustrate this process using the *KAT7* gene detail page.

**Figure 4.**
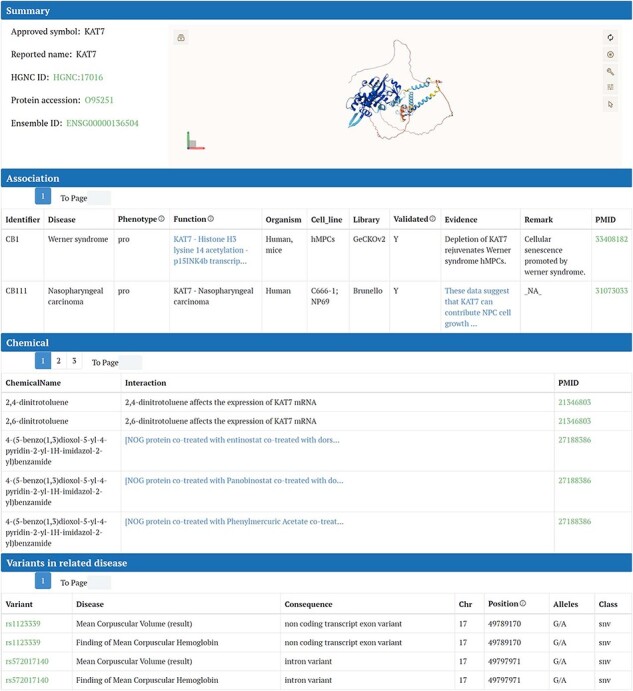
Detail page of CBGDA: Summary section of *KAT7* displays fundamental information and the interactive 3D protein structure, associations between genes and diseases, chemicals for *KAT7*, variants for *KAT7* in related diseases.

The content on this page is organized into several sections. The “Summary” section displays fundamental information about *KAT7*, such as its HGNC ID, UniProt ID, and approved symbol. Adjacent to this basic information, the interactive 3D protein structure of *KAT7* from the Alpha Fold database is presented using the Mol* plugin. All the information regarding the associations between genes and diseases, collected from the article, is displayed in this “Association” section. In the “Chemicals” section, chemicals that interact with or directly influence *KAT7* expression are listed in a table. The first column contains the names of the chemicals, the second column describes their interaction or influence on *KAT7*, and the third column provides the related PubMed ID (Fig. 4). Following the “Chemicals” section, the expression of *KAT7* in cancers and noncancer diseases is presented. For cancer, clicking the “show” button reveals box plots of *KAT7* expression across various types of cancer cell lines (Supplementary Fig. S1). For noncancer diseases, selecting a disease of interest from the dropdown menu and then clicking “show” displays a volcano plot of *KAT7* expression in that disease, with *KAT7* marked on the plot (Supplementary Fig. S2). Both types of plots can be downloaded in PDF format. If the gene is not present in the data downloaded from DepMap or GEN, it will be displayed as “Failed.” In the “Variants” section, the mutation sites of *KAT7* in related diseases are listed, including specific chromosomal location and mutation type (Fig. 4).

#### Disease detail page

For the disease detail page, we used Werner syndrome as an example.

The “Summary” section introduces Werner syndrome and is followed by “External Resources,” which are direct links to authoritative databases or websites for more detailed information on Werner syndrome. The “Chemicals” section lists chemicals that interact with or directly affect the expression of Werner syndrome in a tabulated format. In the “Variants” section, gene mutations caused by Werner syndrome are detailed. The information in this section not only slightly varies from that on the gene page but also includes specifics like chromosomal location. This organization ensures a comprehensive understanding of the disease, its associated mutations, and potential chemical interactions.

#### Download page

We created the download page. The first version (up to September 2023) of the CBGDA data is now ready. Downloadable files including information on gene, disease, chemical, and variant were prepared according to annotation datasets. To meet the users’ needs, we provide datasets in both CSV and TXT formats. If needed, users can select the desired dataset from the dropdown menu above for downloading.

## Discussion and conclusion

The relationship between diseases and genes is intricate and complex. Certain genes have been shown to promote one type of disease while inhibiting another [26], highlighting the need to uncover clues within the interactions between genes and their associated diseases. Such interaction relationships can be found in various database resources. CRISPR, undoubtedly, is the most efficient gene editing technology available today. By utilizing CRISPR technology for genome-wide screening, we can identify genes that are relevant to diseases with greater effectiveness, precision, and reliability compared to other methods and techniques.

In our approach, we specifically collected articles that employed genome-wide CRISPR screening to uncover the association between genes and diseases. We focused on studies that provided clear and definitive evidence of the relationship between genes and diseases and manually annotated the research findings. Leveraging the advantages of CRISPR, we have developed the CBGDA database, which provides a comprehensive collection of key host genes involved in various disease mechanisms, discovered through genome-wide CRISPR screening, along with their associations. The core data in CBGDA are derived from the manual curation of thousands of genome-wide CRISPR-related publications sourced from PubMed. Additionally, external data from authoritative databases and literature are also incorporated. In future iterations, we plan to continually update the database with the latest data and integrate online analysis tools to provide a robust platform for biologists to explore the intricate relationships between genes and diseases.

Our work also has certain limitations. On one hand, the data we have collected focus solely on gene regions identified through CRISPR screening. However, numerous other functional components within the genome have been found to be associated with certain diseases. In fact, the majority of disease-related mutations are located in noncoding regions, such as silencers, insulators, 5ʹ UTR and 3ʹ UTR, and introns [27], all of which can be targeted using CRISPR. On the other hand, the data we have collected may not encompass all relevant publications that have been published. Many more relevant publications could be retrieved by searching full-text articles in PubMed Central. Considering these two aspects, we will adopt new strategies in the future to further enhance and improve the database, ensuring that it becomes more comprehensive and reliable.

Overall, by harnessing the power of CRISPR technology, our database provides a valuable resource for researchers in the field of computational biology, enabling them to delve deeper into the intricate network of gene–disease relationships. This database serves as a foundation for further research and paves the way for advancements in disease diagnosis, prevention, and treatment.

## Supplementary Material

baae077_Supp

## Data Availability

CBGDA is freely accessible at http://cbgda.zhounan.org/main.
