# Supplementary material for: CBGDA: a manually curated resource for gene–disease associations based on genome-wide CRISPR
Source: Database (Oxford). 2024 Aug 30;2024:baae077. doi: 10.1093/database/baae077 (PMC11363955; doi:10.1093/database/baae077)
Supplement: baae077_Supp [file baae077_supp.zip › suppl_data/Source code.pdf]

Models.py

```
from django.db import models

class crispr_template_sheet1(models.Model):

    Gene_symbol = models.CharField(max_length=100)

    Reported_name = models.CharField(max_length=100)

    HGNC_ID = models.CharField(max_length=100)

    UniprotAC = models.CharField(max_length=100)

    Disease_name = models.CharField(max_length=100)

    phenotype = models.CharField(max_length=100)

    function = models.CharField(max_length=255)

    screen_type = models.CharField(max_length=100)

    organism = models.CharField(max_length=100)

    screen_cell_line = models.CharField(max_length=100)

    validated = models.CharField(max_length=100)

    evidence = models.CharField(max_length=10000)

    library_name = models.CharField(max_length=100)

    remark = models.CharField(max_length=10000)

    PMID = models.CharField(max_length=100)

    Ensembl_ID = models.CharField(max_length=100)

    Identifier = models.CharField(max_length=100)

    class Meta:

        db_table = 'crispr_template_sheet1'

class crispr_template_sheet2(models.Model):

    id = models.AutoField(primary_key=True)

    Alias = models.CharField(max_length=255)

    Disease_name = models.CharField(max_length=255)

    description = models.CharField(max_length=10000)

    Disease_Ontology = models.CharField(max_length=100)

    Mesh = models.CharField(max_length=100)

    OMIM = models.CharField(max_length=100)

    ICD10 = models.CharField(max_length=10000)

    ICD10_Class = models.CharField(max_length=100)

    ICD11 = models.CharField(max_length=10000)

    ICD11_Class = models.CharField(max_length=100)

    MCID = models.CharField(max_length=100)

    class Meta:

        db_table = 'crispr_template_sheet2'

class ctd_chem_gene_ixns(models.Model):

    id = models.AutoField(primary_key=True)

    ChemicalName = models.CharField(max_length=255)

    GeneSymbol = models.CharField(max_length=255)

    Interaction = models.CharField(max_length=255)

    PubMedIDs = models.CharField(max_length=255)

    class Meta:
```

```
        db_table = 'ctd_chem_gene_ixns'
class ctd_chemicals_diseases(models.Model):
    id = models.AutoField(primary_key=True)
    ChemicalName = models.CharField(max_length=255)
    DiseaseName = models.CharField(max_length=255)
    DiseaseID = models.CharField(max_length=255)
    PubMedIDs = models.CharField(max_length=255)
    class Meta:
        db_table = 'ctd_chemicals_diseases'
class disease_variants_data(models.Model):
    Disease = models.CharField(max_length=255)
    Variant = models.CharField(max_length=255)
    Gene = models.CharField(max_length=255)
    Chr = models.CharField(max_length=255)
    Position = models.CharField(max_length=255)
    Consequence = models.CharField(max_length=255)
    Alleles = models.CharField(max_length=255)
    Class = models.CharField(max_length=255)
    Alt_Ref_Genome = models.CharField(max_length=255)
    Alt_Ref_Exome = models.CharField(max_length=255)
    class Meta:
        db_table = 'disease_variants_data'
class gene_variants_data(models.Model):
    Disease = models.CharField(max_length=255)
    Variant = models.CharField(max_length=255)
    Gene = models.CharField(max_length=255)
    Chr = models.CharField(max_length=255)
    Position = models.CharField(max_length=255)
    Consequence = models.CharField(max_length=255)
    Alleles = models.CharField(max_length=255)
    Class = models.CharField(max_length=255)
    class Meta:
        db_table = 'gene_variants_data'
```

Views.py

```
import json
import datetime
import os
from django.views.decorators.csrf import csrf_protect, csrf_exempt
from django.http import JsonResponse, HttpResponse, HttpResponseServerError
from django.shortcuts import render
from django.core.files.storage import FileSystemStorage
import app.models
```

```python
import subprocess

os.chdir("app/static/files/")

WORKDIR = os.path.dirname(__file__)

def index(req):

    return render(req, 'index.html')

def main(req):

    return render(req, 'main.html')

def browse(req):

    count1 = app.models.crispr_template_sheet1.objects.count()

    count2 = app.models.crispr_template_sheet2.objects.count()

    page = int(req.GET.get('page', 1))

    sheet = int(req.GET.get('sheet', 1))

    start = (page - 1) * 10

    end = page * 10

    data = []

    if sheet == 1:

        table_data = app.models.crispr_template_sheet1.objects.all().order_by('id')[start:end]

        for item in table_data:

            data.append({

                'Gene_symbol': item.Gene_symbol,

                'Reported_name': item.Reported_name,

                'HGNC_ID': item.HGNC_ID,

                'UniprotAC': item.UniprotAC,

                'Disease_name': item.Disease_name,

                'phenotype': item.phenotype,

                'function': item.function,

                'screen_type': item.screen_type,

                'organism': item.organism,

                'screen_cell_line': item.screen_cell_line,

                'validated': item.validated,

                'evidence': item.evidence,

                'library_name': item.library_name,

                'remark': item.remark,

                'PMID': item.PMID,

                'Identifier': item.Identifier,


            })

    else:

        table_data = app.models.crispr_template_sheet2.objects.all().order_by('id')[start:end]

        for item in table_data:

            data.append({

                'Disease_name': item.Disease_name,

                'Alias': item.Alias,

                'description': item.description,

                'Disease_Ontology': item.Disease_Ontology,
```

```
            'Mesh': item.Mesh,
            'OMIM': item.OMIM,
            'ICD10': item.ICD10,
            'ICD10_Class': item.ICD10_Class,
            'ICD11': item.ICD11,
            'ICD11_Class': item.ICD11_Class,
            'MCID': item.MCID,
        })
    return render(req, 'Browse.html', {"count1": count1, "count2": count2, 'data': data})
def contact(req):
    return render(req, 'contact.html')
def diseaseinfo(req):
    disease = req.GET.get('disease')
    result = app.models.crispr_template_sheet2.objects.filter(Disease_name=disease)
    alias = []
    if result.exists():         alias = [entry.Alias for entry in result]
    alias = alias[0]
    data = []
    sequence = app.models.crispr_template_sheet1.objects.filter(Disease_name=disease)
    count = len(sequence)
    for item in sequence:
        data.append({
                'Gene_symbol': item.Gene_symbol if item.Gene_symbol else '',
                'Disease_name': item.Disease_name if item.Disease_name else '',
                'phenotype': item.phenotype if item.phenotype else '',
                'function': item.function if item.function else '',
                'screen_type': item.screen_type if item.screen_type else '',
                'organism': item.organism if item.organism else '',
                'screen_cell_line': item.screen_cell_line if item.screen_cell_line else '',
                'validated': item.validated if item.validated else '',
                'evidence': item.evidence if item.evidence else '',
                'library': item.library_name if item.library_name else '',
                'remark': item.remark if item.remark else '',
                'PMID': item.PMID if item.PMID else '',
                'Identifier': item.Identifier
        })
    data0 = []
    variants = app.models.disease_variants_data.objects.filter(Disease=alias)
    count0 = len(variants)
    for item in variants:
        data0.append({
            'Disease': item.Disease if item.Disease else '',
            'Variant': item.Variant if item.Variant else '',
            'Gene': item.Gene  if item.Gene else '',
            'Chr': item.Chr if item.Chr else '',
```

```
            'Position': item.Position if item.Position else '',
            'Consequence': item.Consequence if item.Consequence else '',
            'Alleles': item.Alleles if item.Alleles else '',
            'Class': item.Class if item.Class else '',
            'Alt_Ref_Genome': item.Alt_Ref_Genome if item.Alt_Ref_Genome else '',
            'Alt_Ref_Exome': item.Alt_Ref_Exome if item.Alt_Ref_Exome else ''
        })
    return render(req, 'diseaseinfo.html', {'disease': disease, 'data': data, 'data0': data0, 'count': count,
                                            'count0': count0})
def header(req):
    return render(req, 'header.html')
def footer(req):
    return render(req, 'footer.html')
def helps(req):
    return render(req, 'help.html')
def download(req):
    return render(req, 'download.html')
def geneinfo(req):
    gene = req.GET.get('gene')
    page = int(req.GET.get('page', 1))
    data = []
    start = (page - 1) * 10
    end = page * 10
    sequence = app.models.ctd_chem_gene_ixns.objects.filter(GeneSymbol=gene)
    count = len(sequence)
    for item in sequence:
        data.append({
            "ChemicalName": item.ChemicalName,
            "GeneSymbol": item.GeneSymbol,
            "Interaction": item.Interaction,
            "PubMedIDs": item.PubMedIDs
        })
    data0 = []
    variants = app.models.gene_variants_data.objects.filter(Gene__contains=gene)
    count0 = len(variants)
    for item in variants:
        data0.append({
            'Disease': item.Disease if item.Disease else '',
            'Variant': item.Variant if item.Variant else '',
            'Gene': item.Gene if item.Gene else '',
            'Chr': item.Chr if item.Chr else '',
            'Position': item.Position if item.Position else '',
            'Consequence': item.Consequence if item.Consequence else '',
            'Alleles': item.Alleles if item.Alleles else '',
            'Class': item.Class if item.Class else '',
```

```
        })
        data2 = []
        info = app.models.crispr_template_sheet1.objects.filter(Gene_symbol=gene)
        count2 = len(info)
        for item in info:
            data2.append({
                'Disease_name': item.Disease_name if item.Disease_name else '',
                'phenotype': item.phenotype if item.phenotype else '',
                'function': item.function if item.function else '',
                'screen_type': item.screen_type if item.screen_type else '',
                'organism': item.organism if item.organism else '',
                'screen_cell_line': item.screen_cell_line if item.screen_cell_line else '',
                'validated': item.validated if item.validated else '',
                'evidence': item.evidence if item.evidence else '',
                'library': item.library_name if item.library_name else '',
                'remark': item.remark if item.remark else '',
                'PMID': item.PMID if item.PMID else '',
                'Identifier': item.Identifier,
            })
    return render(req, 'geneinfo.html', {'gene': gene, 'data': data, 'count': count,
                                         'data0': data0, 'count0': count0,
                                         'data2': data2, 'count2': count2})
def search_browse(req):
    search_text = req.GET.get('text')
    data = []
    if search_text:
        from_Gene_symbol = app.models.crispr_template_sheet1.objects.filter(Gene_symbol__icontains=search_text)
        if from_Gene_symbol:
            sequence = app.models.crispr_template_sheet1.objects.values_list('id', flat=True)
            for item in from_Gene_symbol:
                data.append({
                    'Gene_symbol': item.Gene_symbol,
                    'Disease_name': item.Disease_name,
                    'record': list(sequence.values_list('id', flat=True)).index(item.id),
                    'sheet': 1,
                    'Reported_name': item.Reported_name,
                    'HGNC_ID': item.HGNC_ID,
                    'UniprotAC': item.UniprotAC,
                    'phenotype': item.phenotype,
                    'function': item.function,
                    'screen_type': item.screen_type,
                    'organism': item.organism,
                    'screen_cell_line': item.screen_cell_line,
                    'validated': item.validated,
                    'evidence': item.evidence,
```

```
                'library_name': item.library_name,
                'remark': item.remark,
                'PMID': item.PMID,
                'Identifier': item.Identifier,
            })
        from_Disease_name = app.models.crispr_template_sheet2.objects.filter(Disease_name__icontains=search_text)
        if from_Disease_name:
            sequence = app.models.crispr_template_sheet2.objects.values_list('id', flat=True)
            for item in from_Disease_name:
                data.append({
                    'Gene_symbol': '',
                    'Disease_name': item.Disease_name,
                    'record': list(sequence.values_list('id', flat=True)).index(item.id),
                    'sheet': 2
                })
        if data:
            return JsonResponse(data, safe=False)
        else:
            return JsonResponse("Entry not found.", safe=False)
    else:
        return JsonResponse("Entry not found.", safe=False)
def gene_sum(req):
    search_text = req.GET.get('text')
    data = []
    sequence = app.models.crispr_template_sheet1.objects.filter(Gene_symbol=search_text)
    for item in sequence:
        data.append({
            'Gene_symbol': item.Gene_symbol,
            'Disease_name': item.Disease_name,
            'HGNC_ID': item.HGNC_ID,
            'Reported_name': item.Reported_name,
            'UniprotAC': item.UniprotAC,
            'Ensembl_ID': item.Ensembl_ID,
        })
    if data:
        return JsonResponse(data, safe=False)
    else:
        return JsonResponse("Entry not found.", safe=False)
def dise_sum(req):
    search_text = req.GET.get('text')
    data = []
    sequence = app.models.crispr_template_sheet2.objects.filter(Disease_name=search_text)
    for item in sequence:
        data.append({
            'Disease_name': item.Disease_name,
```

```
            'Alias': item.Alias,
            'description': item.description,
            'Disease_Ontology': item.Disease_Ontology,
            'Mesh': item.Mesh,
            'OMIM': item.OMIM,
            'ICD10': item.ICD10,
            'ICD10_Class': item.ICD10_Class,
            'ICD11': item.ICD11,
            'ICD11_Class': item.ICD11_Class,
            'MCID': item.MCID,
        })
    if data:
        return JsonResponse(data, safe=False)
    else:
        return JsonResponse("Entry not found.", safe=False)
def geneinfo_r(req):
    gene = req.GET.get('gene')
    disease = req.GET.get('disease')
    r_script_path = os.path.join(WORKDIR, 'static/files/expression_analysis.R')
    r_command = f'Rscript {r_script_path} "{gene}" "{disease}"'
    try:
        subprocess.check_output(r_command, shell=True)
        return HttpResponse('Done')
    except subprocess.CalledProcessError as e:
        return HttpResponse('Failed')
def volcano_r(req):
    gene = req.GET.get('gene')
    disease = req.GET.get('disease')
    print('gene:'+gene+';'+'disease:'+disease)
    r_script_path = os.path.join(WORKDIR, 'static/files/volcano.R')
    r_command = f'Rscript {r_script_path} "{gene}" "{disease}"'
    try:
        subprocess.check_output(r_command, shell=True)
        return HttpResponse('Done')
    except subprocess.CalledProcessError as e:
        return HttpResponse('Failed')
def download_view(request):
    if request.method == 'POST':
        table_name = request.POST.get('table_name')
        format = request.POST.get('format')
        file_name = f'{table_name}.{format}'
        file_path = os.path.join(WORKDIR, 'static/files/' + file_name)
        print(file_path)
        if format == 'txt':
            csv_file_path = os.path.join(WORKDIR, 'static/files/' + f'{table_name}.csv').replace('\\', '/')
```

```
            txt_file_path = file_path

            convert_csv_to_txt(csv_file_path, txt_file_path)

            file_name = f'{table_name}.txt'

            file_path = txt_file_path

        response = HttpResponse(content_type='application/octet-stream')

        response['Content-Disposition'] = f'attachment; filename="{file_name}"'

        with open(file_path, 'rb') as file:

            response.write(file.read())

        return response

    table_names = [

        'CBGDA_chem_disease2309',

        'CBGDA_chem_gene2309',

        'CBGDA_main_2309',

        'CBGDA_variants_disease2309',

        'CBGDA_variants_gene2309',

    ]

    return render(request, 'download.html', {'table_names': table_names})

def convert_csv_to_txt(csv_file_path, txt_file_path):

    with open(csv_file_path, 'r') as csv_file, open(txt_file_path, 'w') as txt_file:

        for line in csv_file:

            txt_file.write(line.replace(',', '\t'))
```
