# Supplementary figures and images for: CBGDA: a manually curated resource for gene–disease associations based on genome-wide CRISPR
Source: Database (Oxford). 2024 Aug 30;2024:baae077. doi: 10.1093/database/baae077 (PMC11363955; doi:10.1093/database/baae077)

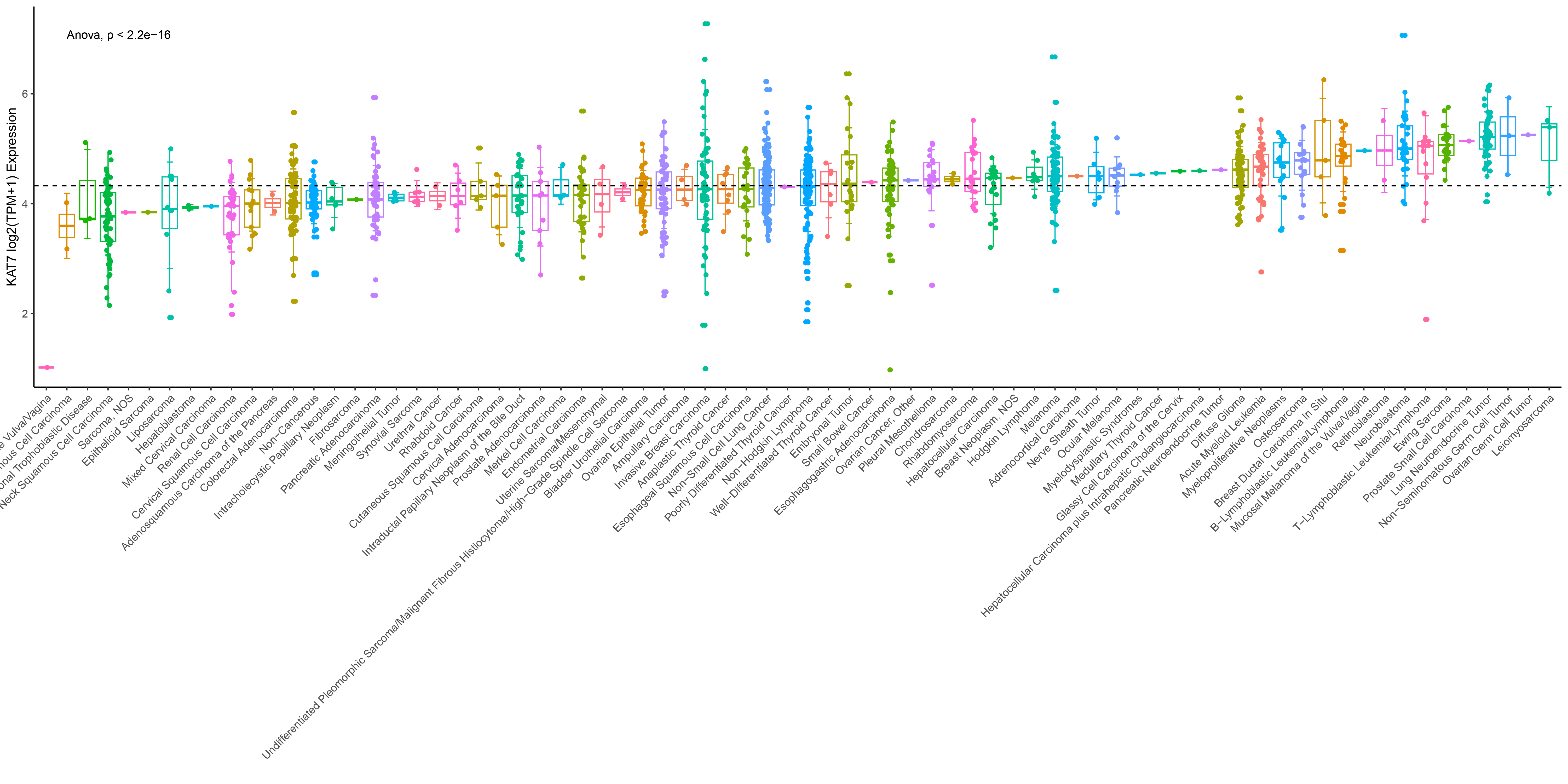

Supplement: baae077_Supp [file baae077_supp.zip › suppl_data/Supplemental Figure 1.png]

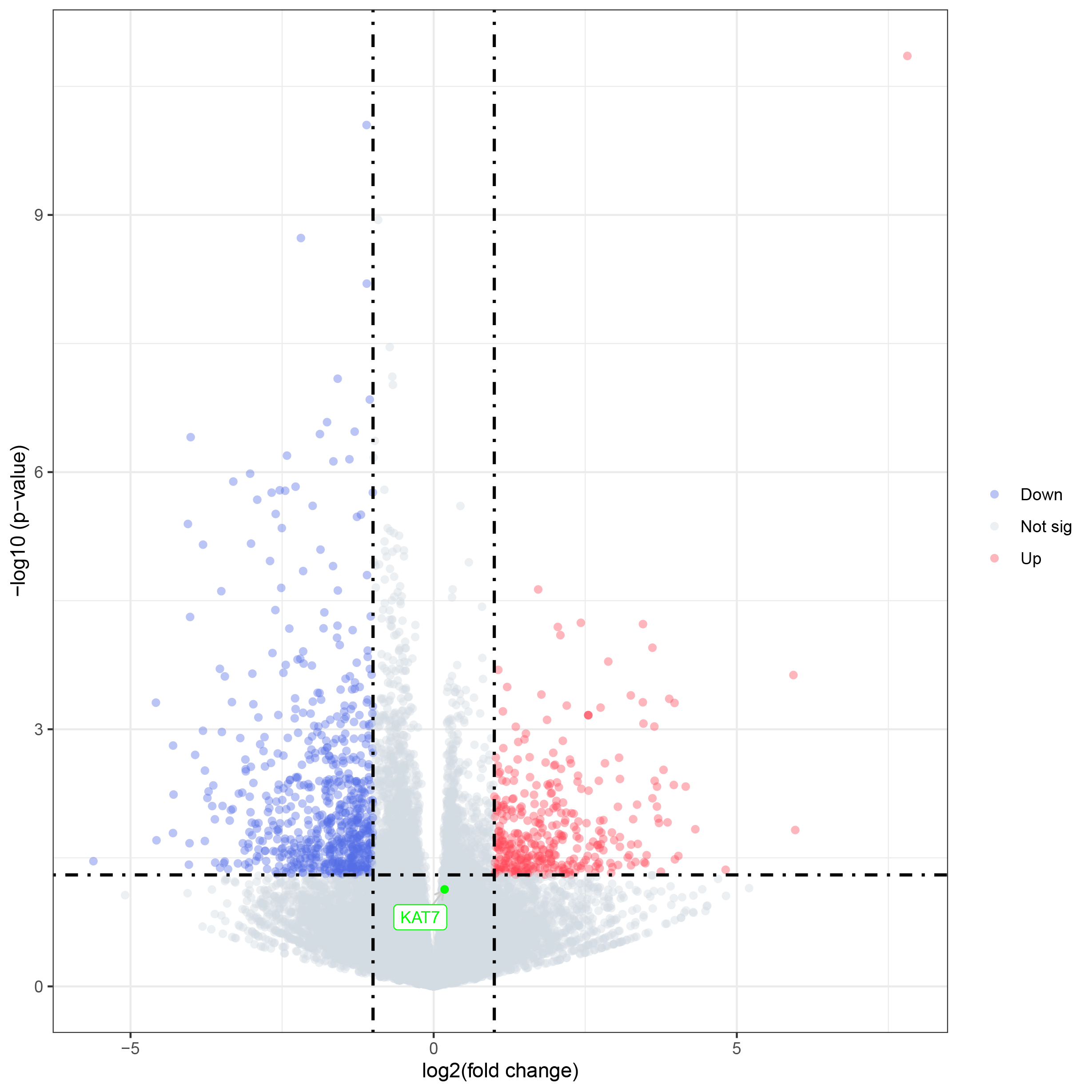

Supplement: baae077_Supp [file baae077_supp.zip › suppl_data/Supplemental Figure 2.png]
